# Messenger RNA vaccines and beyond: Molecular design, delivery platforms, and emerging therapeutic applications

**DOI:** 10.1016/j.omtn.2026.103140

**Published:** 2026-10-14

**Authors:** Kyung-Soo Lee, Jun-Young Park, Chang-Ung Kim

**Affiliations:** 1 College of Pharmacy, Chosun University, Gwangju 61452, Republic of Korea; 2 Research Institute of Pharmaceutical Sciences (RIPS), College of Pharmacy, Chosun University, Dong-gu, Gwangju 61452, Republic of Korea; 3 Environmental Diseases Research Center, Korea Research Institute of Bioscience and Biotechnology (KRIBB), Daejeon 34141, Republic of Korea; 4 Honam Regional Future Drug Development Convergence Education & Research Division (BK21 FOUR), Chosun University, Dong-gu, Gwangju 61452, Republic of Korea

**Keywords:** MT: Delivery Strategies, mRNA vaccines, mRNA therapeutics, lipid nanoparticles, transcript engineering, RNA delivery, self-amplifying RNA, circular RNA, genome editing

## Abstract

Messenger RNA (mRNA) medicine has progressed from a conceptual nucleic acid modality to a clinically validated platform for transient protein expression, with SARS-CoV-2 vaccines providing population-scale proof of maturity. This review synthesizes the molecular and translational principles underlying this transition, emphasizing two determinants of mRNA performance: transcript engineering and delivery formulation. Transcript engineering integrates cap structure, untranslated regions, coding-sequence optimization, nucleoside chemistry, and poly(A) design, whereas formulation controls encapsulation, tissue tropism, cytosolic release, stability, and route-dependent biodistribution. We examine how this platform logic extends beyond vaccination to protein replacement, lung-directed gene complementation, regenerative signaling, *in vivo* antibody production, local tumor immunomodulation, genome editing, and transient *ex vivo* cell engineering. We also compare conventional mRNA, self-amplifying RNA, and circular RNA as complementary architectures with distinct dose requirements, expression durability, immunogenicity, and manufacturability. Across preventive and therapeutic settings, translation is constrained by extrahepatic delivery specificity, productive endosomal escape, innate and carrier-driven immune responses during repeat dosing, and scalable manufacturing with robust analytical quality control. The future of mRNA medicine will depend on platform matching: aligning RNA architecture, formulation, administration route, and disease context with clinical settings in which transient expression offers a genuine therapeutic advantage.

## Graphical abstract

**Figure:**
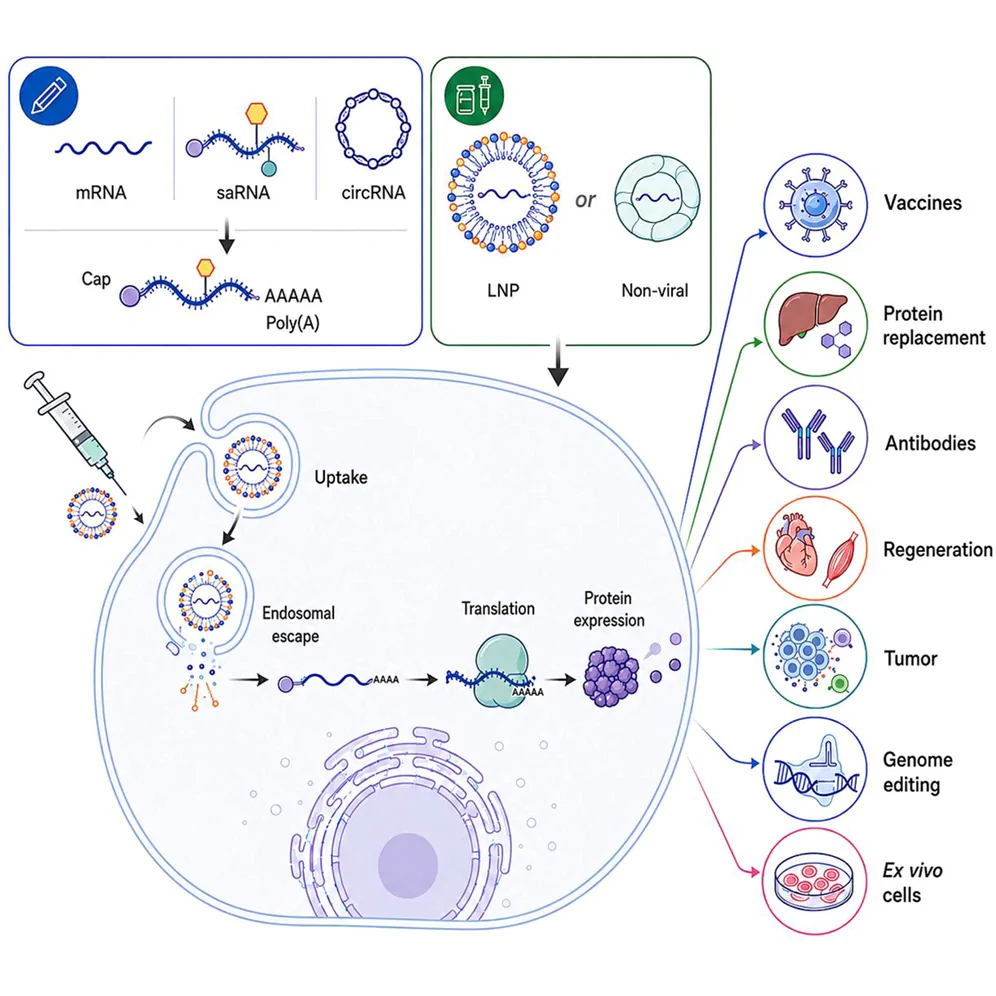

## Teaser

Kim and colleagues review mRNA medicine as a programmable transient protein-expression platform, emphasizing how transcript architecture, lipid nanoparticle delivery, next-generation RNA formats, and disease-specific platform matching shape therapeutic applications beyond vaccination.

## Introduction

Messenger RNA (mRNA) vaccines have evolved from a conceptual form of nucleic acid immunization into a clinically validated biomedical platform.^1^^,^^2^^,^^3^ Foundational studies established that exogenous mRNA could drive protein expression *in vivo* and, when appropriately delivered, induce antigen-specific cellular and humoral immune responses.^4^^,^^5^^,^^6^ Before the COVID-19 pandemic, studies targeting pathogens such as influenza and Zika virus further showed that sequence-engineered mRNA vaccines could induce potent immune responses in animal models and early human studies.^7^^,^^8^ Most decisively, however, the COVID-19 pandemic transformed mRNA vaccines from a promising experimental approach into a technology validated at population scale.

Across influenza, Zika virus, and SARS-CoV-2 programs, distinct antigen sequences have been implemented in related mRNA design and delivery strategies, illustrating the sequence programmability of the platform.^2^^,^^3^^,^^7^^,^^8^ This programmability enables rapid redesign of the encoded payload, while practical performance still depends on transcript engineering, delivery, and control of innate immune sensing.^9^^,^^10^^,^^11^ In this respect, nucleoside modification represented a critical turning point, as it markedly reduced pattern-recognition receptor activation and enabled improved translation and biological performance of synthetic mRNA.^9^^,^^12^

Importantly, the significance of the mRNA platform now extends beyond vaccination alone. Primary clinical studies have demonstrated that mRNA can be redirected beyond antigen expression toward enzyme replacement, *in vivo* monoclonal antibody production, and transient delivery of genome-editing machinery.^13^^,^^14^^,^^15^ From this perspective, mRNA vaccines should be understood not as an isolated vaccine modality but as the first broadly successful clinical entry point into a larger class of programmable mRNA medicines.

Recent reviews have already provided broad surveys of non-vaccine mRNA applications, targeted delivery strategies, and the clinical development of protein-replacement, antibody-encoding, cancer-immunotherapy, and genome-editing programs.^16^^,^^17^^,^^18^ Accordingly, the purpose of the present review is not to provide another application-centered catalog of mRNA products. Instead, we use the clinical maturation of mRNA vaccination as a reference point to address a more specific translational question of which combinations of RNA architecture, delivery system, administration route, target tissue, expression kinetics, and repeat-dose requirements allow transient protein expression to provide a genuine therapeutic advantage.

The distinguishing scope of this review is therefore a platform-matching framework derived from comparative analysis of primary mechanistic, preclinical, and clinical evidence across therapeutic applications. This framework connects molecular design and manufacturing quality with the biological requirements of individual therapeutic applications. Rather than treating changes in the encoded sequence as sufficient to redirect a common platform, we compare how delivery accessibility, required expression magnitude and duration, local versus systemic administration, and repeat-dose feasibility shape clinical performance across vaccination, protein replacement, regenerative signaling, *in vivo* antibody production, local tumor immunomodulation, genome editing, and transient cell engineering. This framework also evaluates conventional mRNA, self-amplifying RNA (saRNA), circular RNA (circRNA), and alternative non-viral carriers as complementary rather than interchangeable solutions, distinguishes approved and late-stage clinical programs from early proof-of-concept studies, and organizes the remaining barriers around tissue-selective delivery, productive cytosolic release, carrier-associated immunity, and scalable analytical manufacturing. Figure 1 summarizes this design-to-expression framework and its relationship to therapeutic outcome.

**Figure 1:**
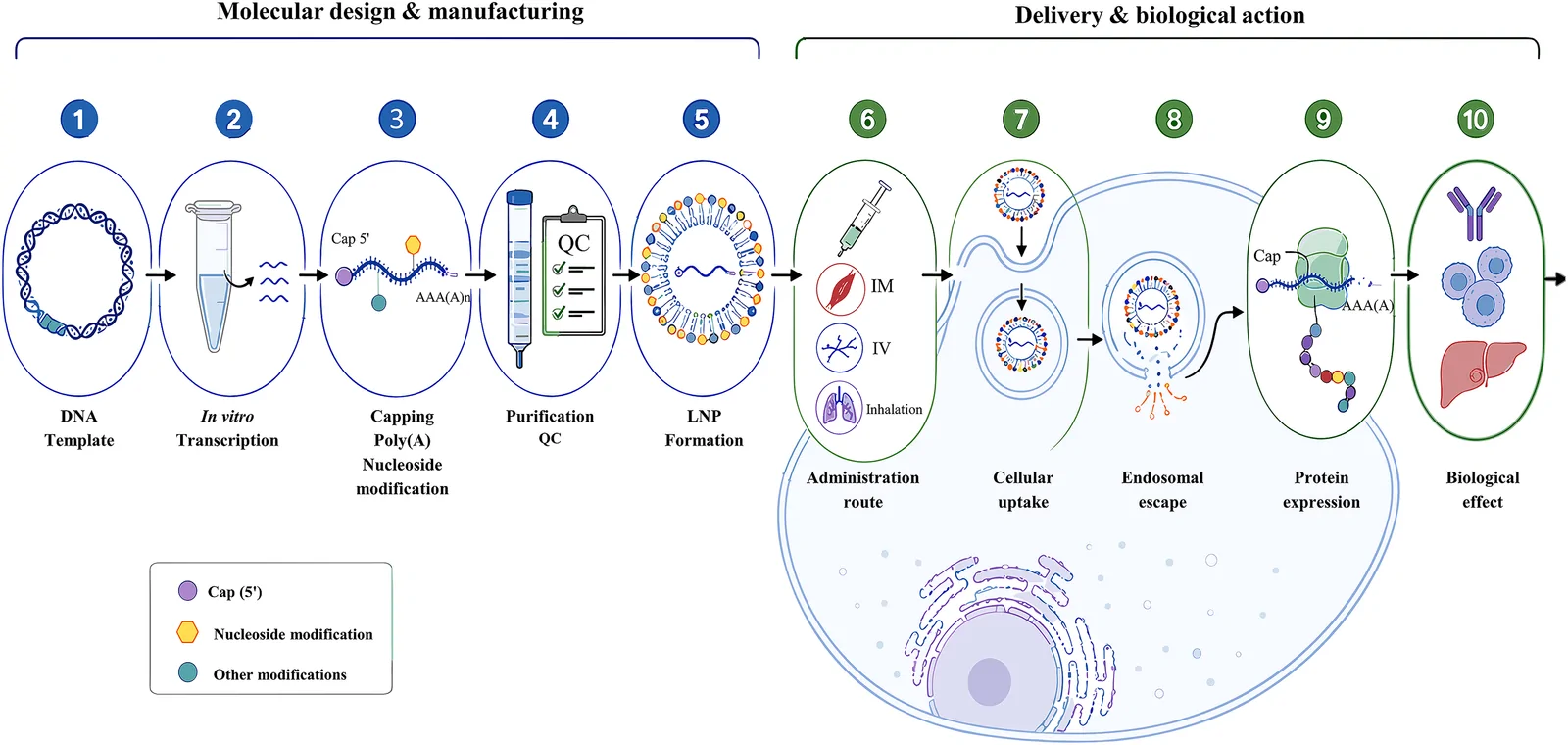
mRNA therapeutic design-to-expression pipeline The schematic summarizes the sequential relationship between transcript design, manufacturing, formulation, delivery, endosomal escape, cytosolic translation, and downstream biological activity. This schematic figure was prepared based on author-provided conceptual sketches and scientific layout, using FigureLab for scientific illustration and manual author editing.

## Evolution of mRNA vaccine technology: From molecular concept to clinical platform

### Establishing mRNA as a programmable biological template

The discovery of mRNA was a cumulative process involving conceptual and experimental contributions from several laboratories rather than a single event.^19^ In the mid-1950s, Volkin and Astrachan identified rapidly synthesized RNA in bacteriophage-infected *Escherichia coli*, and subsequent work demonstrated its rapid turnover, providing early evidence for a transient RNA population.^20^^,^^21^ In 1961, two complementary studies published in the same issue of *Nature*—by Brenner, Jacob, and Meselson and by Gros and colleagues—demonstrated the existence of short-lived RNA that carries genetic information to ribosomes, thereby establishing the experimental basis for the mRNA concept.^22^^,^^23^ Much later, cationic liposomes were shown to mediate RNA transfection in cultured cells, establishing that exogenous RNA could be delivered experimentally rather than merely observed as a biological intermediate.^24^ This delivery concept became therapeutically relevant when direct injection of *in*-*vitro*-transcribed RNA into mouse muscle drove *in vivo* protein expression, demonstrating that RNA itself could function as a transient expression platform.^4^ Shortly thereafter, liposome-entrapped mRNA was shown to induce virus-specific cytotoxic T-lymphocyte responses in mice, providing one of the earliest demonstrations that synthetic mRNA could serve as a vaccine modality.^5^ In parallel, self-replicating Semliki Forest virus RNA was developed as a recombinant vaccine platform, anticipating later saRNA strategies designed to reduce dose requirements and prolong antigen expression.^6^

### Early translational challenges in synthetic mRNA delivery

Although early proof-of-concept studies established that exogenous mRNA could drive protein expression and function as a vaccine platform, clinical translation remained limited because synthetic and process-derived RNA triggered innate immune sensing that suppressed productive expression and raised safety concerns.^9^^,^^25^^,^^26^ A key turning point came when Karikó and colleagues showed that unmodified RNA activates human Toll-like receptor (TLR)3, TLR7, and TLR8, thereby reframing the central problem of the field: successful mRNA therapeutics would require not only efficient delivery but also active control of RNA immunogenicity.^9^ This insight shifted the field from proof of expression alone toward the engineering of immune-compatible transcripts and cleaner manufacturing workflows.

### Engineering immune-compatible mRNA through nucleoside modification

The decisive inflection point in the field came from nucleoside modification, which transformed synthetic mRNA from a highly inflammatory molecule into a more translationally competent therapeutic format.^9^ Subsequent comparative studies showed that N1-methylpseudouridine (m1Ψ)-containing mRNA can outperform pseudouridine-containing mRNA by providing higher protein expression and lower immunogenicity in mammalian systems.^12^ Later work further demonstrated that the immune-activating profile of therapeutic mRNA is shaped not only by nucleoside chemistry but also by manufacturing quality, particularly the reduction of double-stranded RNA (dsRNA) impurities, underscoring that clinically viable mRNA depends on process control as much as on sequence design.^10^^,^^11^

### COVID-19 vaccines as a proof of platform maturity

The COVID-19 pandemic provided the first full-scale clinical validation of the platform, with early human testing of mRNA-1273 showing rapid induction of binding and neutralizing antibody responses after vaccination.^27^ This translational momentum culminated in pivotal phase 3 trials showing 95.0% efficacy for BNT162b2 and 94.1% efficacy for mRNA-1273 against symptomatic COVID-19 in the pre-Omicron setting.^2^^,^^3^ Accordingly, the historical significance of the pandemic was not simply that two products succeeded but that mRNA vaccines were validated as a rapid, programmable, and scalable medical platform.^2^^,^^3^^,^^27^

## Molecular design and mechanistic principles of mRNA vaccines

### Cellular delivery, antigen expression, and immune priming

The central mechanism of mRNA vaccination is based on transient intracellular expression of an antigen encoded by synthetic mRNA. Following administration, productive delivery requires the RNA cargo to reach the cytoplasm, where translation occurs. *In vivo* imaging of nucleoside-modified mRNA-lipid nanoparticles (LNPs) has demonstrated route-dependent local and systemic protein expression, with intravenous administration producing prominent hepatic expression.^28^ Productive intracellular delivery is further constrained by endosomal escape, and structure-activity optimization of ionizable lipids has directly linked improved cytosolic release with enhanced mRNA pharmacology in rodents and non-human primates.^29^ Once in the cytoplasm, the delivered mRNA serves as a template for protein synthesis. Foundational *in vivo* experiments demonstrated that direct administration of mRNA could produce the encoded protein in mammalian tissue.^4^ The immunological consequences of this expression depend on which cells produce or acquire the encoded antigen and how antigen expression is coupled to antigen presentation and innate immune activation. Recent mechanistic studies indicate that mRNA-LNP vaccination engages multiple antigen-presenting cell (APC) pathways rather than relying on a single canonical route. Jo et al. demonstrated that conventional dendritic cell (cDC)1 and cDC2 contribute redundantly to CD8+ T cell priming after mRNA-LNP vaccination and further identified acquisition of peptide-MHC class I complexes through cross-dressing as a substantial route of antigen presentation.^30^ Complementary work by Kubara et al. showed that cDCs function as the principal antigen-presenting population in their mRNA-LNP model, whereas macrophages in draining lymph nodes promote innate immune activation and APC maturation through STING-type I interferon signaling.^31^ Mechanistic analysis of BNT162b2 further showed that vaccine mRNA was preferentially detected in monocyte/macrophage and dendritic cell populations in draining lymph nodes and that the resulting CD8+ T cell response depended on MDA5 and type I interferon signaling.^32^ Primary human studies further demonstrate how these early events develop into coordinated adaptive immunity. Following SARS-CoV-2 mRNA vaccination, antigen-specific CD4+ T cell responses were rapidly induced after priming, whereas CD8+ T cell responses developed more gradually; early Th1 and T follicular helper responses were associated with subsequent CD8+ T cell and neutralizing antibody responses, respectively.^33^ Direct sampling of draining lymph nodes demonstrated persistent antigen-specific germinal center B cell responses after mRNA vaccination.^34^ Longitudinal analyses further showed that functional antigen-specific memory B cells increased between 3 and 6 months after vaccination and were accompanied by durable antigen-specific CD4+ and CD8+ T cell responses.^35^ Collectively, these primary findings indicate that mRNA-vaccine immunogenicity reflects the integration of productive intracellular delivery and antigen expression with APC engagement, innate immune signaling, and the development of cellular and humoral immunity.

Importantly, transient protein expression from a cytoplasmic mRNA template, rather than vaccine-specific immune priming itself, provides the common mechanistic link between mRNA vaccination and broader therapeutic applications. Primary clinical studies demonstrate that this expression principle has already been implemented across distinct therapeutic settings, including enzyme replacement with mRNA-3927,^15^ *in vivo* monoclonal antibody production with mRNA-1944,^13^ intratumoral immunomodulation with mRNA-2752,^36^ and transient Cas9 expression for *in vivo* genome editing with NTLA-2001.^14^ The biological consequence of this common expression principle is nevertheless application dependent, being determined by the identity of the encoded protein, the cells and tissues reached by the delivery system, and the required magnitude, localization, and duration of expression. Accordingly, in the subsequent sections, we evaluate these non-vaccine applications as distinct therapeutic implementations of a common mRNA expression principle rather than as interchangeable extensions of the vaccine platform.

### Structural architecture of synthetic mRNA

Therapeutic and vaccine mRNAs are typically engineered as linear transcripts containing a 5′ cap, a 5′ untranslated region (UTR), an open reading frame, a 3′ UTR, and a poly(A) tail. These elements are not merely structural components, as their sequence and configuration directly influence translational output and transcript persistence. Experiments with synthetic cap analogs demonstrated that cap chemistry, orientation, and affinity for eIF4E can substantially affect the translational efficiency of *in*-*vitro*-transcribed mRNA.^37^ The 5′ cap and poly(A) tail also function cooperatively, as direct comparison of capped and uncapped, polyadenylated and non-polyadenylated transcripts demonstrated synergistic enhancement of translation.^38^ UTR composition provides an additional layer of control over mRNA performance. Combinatorial screening of 5′ and 3′ UTRs in exogenously delivered therapeutic mRNA demonstrated substantial differences in protein output, with 5′ UTR identity exerting a particularly strong effect in that experimental system.^39^ Consistent with this principle, tandem β-globin 3′ UTRs and extension of the poly(A) tail to 120 nt independently increased the stability and translational efficiency of antigen-encoding RNA.^40^ Translation initiation is additionally sensitive to sequence context around the AUG start codon; site-directed mutagenesis directly demonstrated that individual flanking nucleotides can markedly alter translational efficiency.^41^ The open reading frame determines not only protein identity but also transcript stability, translational kinetics, and protein quality through synonymous sequence design. Codon optimization should therefore not be reduced to maximizing the frequency of host-preferred codons. Synonymous substitutions can alter codon optimality and RNA secondary structure, both of which can influence mRNA stability and protein expression.^42^^,^^43^ In engineered mRNAs, increased coding-region secondary structure was associated with longer functional mRNA half-life and higher protein output.^42^ Indeed, protein expression in systematically designed mRNA variants correlated more strongly with functional mRNA half-life than with instantaneous translation rate, demonstrating that sequence optimization cannot be evaluated using codon adaptation alone.^42^ Moreover, extensive synonymous recoding can alter translational kinetics and protein conformation; codon optimization of coagulation factor IX increased expression but also produced detectable conformational differences in the encoded protein.^44^ Therapeutic mRNA sequence design should therefore be considered a multi-objective optimization problem that balances codon usage, RNA structure, transcript persistence, and protein integrity rather than maximizing any single sequence metric. More broadly, systematic analysis of diverse therapeutic mRNAs demonstrated that intracellular mRNA stability can be a stronger determinant of protein output than ribosome loading alone and that sequence- and structure-based design can simultaneously improve stability and expression.^45^ Modified nucleosides are discussed further below as an additional determinant of translational output and immune compatibility. Taken together, these primary studies support a model in which mRNA architecture functions as a coupled design space rather than as a collection of independent optimization variables. The relevant therapeutic objective is therefore not maximal translation alone but an application-specific balance among expression magnitude, functional persistence, protein integrity, and immune compatibility.

### Tuning innate immune sensing and immunogenicity

Synthetic mRNA is sensed by innate immune pathways because specific molecular features of the transcript, as well as process-derived impurities, can be recognized by endosomal and cytosolic RNA sensors.^9^^,^^11^^,^^25^^,^^26^ Unmodified uridine-rich RNA can stimulate endosomal TLRs, whereas uncapped or incompletely capped 5′-triphosphate RNA and dsRNA contaminants can engage cytosolic sensors such as RIG-I and MDA5, leading to type I interferon signaling.^9^^,^^25^^,^^26^ These sensing events can further activate effector pathways such as PKR and OAS/RNase L, thereby suppressing translation or promoting RNA cleavage.^46^^,^^47^ Functionally, this innate recognition is context dependent rather than uniformly detrimental. In vaccination, MDA5-type I interferon signaling can contribute to CD8+ T cell priming, whereas stronger RNA-driven innate activation can reduce productive protein expression.^11^^,^^32^ The design objective is therefore not complete immunologic silence but application-specific calibration of RNA sensing. In practice, this calibration is achieved through cap engineering, nucleoside modification, sequence optimization, purification to remove dsRNA impurities, and formulation design.^9^^,^^10^^,^^11^ Innate immune control should thus be understood as a functional design variable that governs the balance among protein expression, adjuvant effect, tolerability, and repeat-dose feasibility.^1^^,^^11^ Taken together, these findings indicate that innate immune control is an application-dependent design variable that balances productive expression with the degree of immune stimulation required for the intended use.

### Critical quality attributes of therapeutic mRNA

The clinical performance of mRNA vaccines depends not only on molecular design but also on critical quality attributes that determine identity, purity, potency, stability, and batch consistency.^48^ Capping efficiency is a key quality attribute because cap structure and orientation directly affect the translational efficiency of *in*-*vitro*-transcribed mRNA.^37^ Poly(A) tail length and transcript integrity are also important determinants of functional output; experimental extension of the poly(A) tail increased transcript stability and translational efficiency in antigen-encoding RNA.^40^ Residual dsRNA is one of the most important process-related impurities because it can activate innate immune pathways and suppress productive translation.^10^^,^^11^ Removal of dsRNA by chromatographic purification was shown to reduce immune activation and improve translation of nucleoside-modified protein-encoding mRNA.^10^ Other relevant quality attributes include residual DNA template, free nucleotides, abortive transcripts, endotoxin, RNA integrity, encapsulation efficiency, particle size distribution, and formulation stability.^48^ Primary studies showing that RNA purification and manufacturing processes can alter innate immune activation and protein expression^10^^,^^11^ and that LNP composition can substantially alter *in vivo* mRNA potency^49^ support evaluating RNA and formulation attributes together rather than independently. As the field expands from vaccines to protein replacement, antibody expression, and genome editing, the relative priority of individual quality attributes will likewise become increasingly application specific, reflecting differences in dose, administration route, target tissue, and required expression duration.

## Delivery platforms and formulation strategies

### LNP composition and functional roles

Efficient mRNA delivery requires a carrier system that protects RNA from extracellular degradation, promotes cellular uptake, and enables release of the transcript into the cytoplasm.^50^ LNPs have become the dominant non-viral delivery platform for clinical mRNA vaccines because they combine RNA encapsulation, nanoscale assembly, manufacturability, and transient intracellular delivery.^1^

A typical mRNA-LNP formulation contains four major lipid components: an ionizable lipid, a helper phospholipid, cholesterol, and a polyethylene glycol-conjugated lipid.^51^ The ionizable lipid is the functional core of most modern mRNA-LNP systems because it can remain relatively neutral at physiological pH while becoming protonated under acidic formulation or endosomal conditions.^50^ During formulation, ionizable lipids facilitate electrostatic complexation with the negatively charged mRNA backbone, thereby promoting encapsulation and particle assembly.^52^ After endocytosis, protonation of the ionizable lipid in the acidic endosomal environment promotes interactions with anionic endosomal membrane lipids and contributes to membrane destabilization and endosomal escape.^50^ Helper phospholipids, such as DSPC or related structural lipids, contribute to membrane organization and support the structural integrity of the nanoparticle.^51^ Cholesterol improves particle stability, modulates lipid packing, and can influence membrane fusion behavior and intracellular trafficking.^51^ PEG-lipids reduce nonspecific aggregation, help control particle size during formulation, and improve colloidal stability during storage, although excessive PEG shielding may also reduce cellular uptake or alter biodistribution.^52^ Thus, mRNA-LNP performance is not determined by a single lipid species but by the integrated physicochemical behavior of the entire formulation.^51^ Small changes in lipid chemistry, molar ratio, particle size, surface charge, or buffer composition can substantially affect encapsulation efficiency, potency, tolerability, biodistribution, and storage stability.^52^ These formulation variables have also been demonstrated experimentally rather than only inferred from comparative platform analyses. Factorial optimization of lipid composition and lipid:mRNA ratios increased the *in vivo* potency of an mRNA-LNP formulation by approximately 7-fold, demonstrating that formulation composition itself is a major determinant of mRNA output.^49^ Similarly, systematic optimization of ionizable lipid chemistry improved endosomal escape and supported sustained mRNA pharmacology and tolerability in rodents and non-human primates.^29^

### Endosomal uptake and productive cytoplasmic release

Most mRNA-LNPs enter cells through endocytic pathways rather than by direct fusion with the plasma membrane.^50^ After internalization, the LNP must release mRNA into the cytoplasm because ribosomal translation occurs in the cytosolic compartment.^1^ Therefore, delivery efficiency is not simply cellular uptake but productive cytosolic release of intact mRNA.^50^ Endosomal escape remains one of the major bottlenecks in LNP-mediated nucleic acid delivery.^53^ Studies of LNP-mediated RNA delivery have shown that a substantial fraction of internalized nanoparticles can remain trapped in endosomal compartments or be recycled or degraded rather than releasing their cargo productively into the cytosol.^53^^,^^54^ This explains why high cellular uptake does not necessarily translate into high protein expression or therapeutic potency.^54^ Ionizable lipids are central to endosomal escape because their protonation in acidified endosomes promotes electrostatic interactions with anionic lipids in the endosomal membrane.^50^ These interactions can induce membrane destabilization, non-bilayer lipid structures, or transient membrane defects that permit mRNA release into the cytoplasm.^52^ However, increasing endosomal disruption can also increase inflammatory responses or cytotoxicity, making endosomal escape a formulation problem that must be balanced against safety.^51^ For vaccine applications, relatively modest cytosolic mRNA release may be sufficient if antigen expression and innate immune activation are appropriately balanced.^1^ For protein replacement, antibody expression, or genome editing, however, the required level, location, and duration of expression may be more demanding, making endosomal escape and cytosolic delivery even more critical determinants of clinical performance.^50^

### Tissue tropism and route of administration

The route of administration strongly influences which cells encounter the mRNA-LNP formulation, which antigen-presenting populations are activated, and which tissues contribute to protein expression.^1^ Intramuscular injection is well suited for prophylactic vaccination because it supports local antigen expression and immune cell recruitment while remaining technically simple and clinically scalable.^1^ The clinical success of the BNT162b2 and mRNA-1273 vaccines demonstrated that intramuscular delivery can generate robust protective immunity at population scale.^2^^,^^3^ Intravenous administration produces a distinct biodistribution profile and commonly favors hepatic delivery for many LNP systems.^50^ This liver tropism can be advantageous for applications in which hepatocytes are the intended target, such as liver-directed protein expression or genome editing.^52^ However, extrahepatic mRNA delivery is no longer limited to nonspecific adjustment of particle size, charge, or administration route. Discovery-driven approaches have enabled functional rather than purely biodistribution-based screening of large nanoparticle libraries. For example, the barcoded FIND platform identified LNPs, including 7C2 and 7C3, that delivered mRNA and genome-editing cargo to pulmonary or splenic endothelial populations after systemic administration.^55^ Composition-driven endogenous targeting has also expanded substantially. Selective organ targeting (SORT) LNPs incorporate a supplemental lipid-like component that can redirect functional mRNA delivery from the liver toward the lungs or spleen.^56^ Mechanistic studies indicate that the chemical identity of the SORT component alters apparent pKa, PEG-lipid desorption, serum-protein adsorption, and subsequent receptor-mediated uptake through the formation of organ-associated protein coronas.^57^^,^^58^ Rational modification of ionizable lipid architecture can produce similar extrahepatic tropism without an externally conjugated targeting ligand. Amide-containing N-series lipids and sulfonium-based ionizable lipids have, for example, enabled lung-selective mRNA expression after intravenous administration in mice.^59^^,^^60^ More recent strategies use peptide-encoded surface engineering or peptide-ionizable lipids to program organ-selective protein-corona formation and direct mRNA delivery to tissues including the lungs, spleen, thymus, and bone.^61^^,^^62^

Cell-level targeting can be superimposed on organ-level biodistribution by functionalizing nanoparticles with ligands that bind internalizing cell-surface receptors. CD5-targeted LNPs delivered CAR-encoding mRNA to T lymphocytes and generated transient antifibrotic CAR T cells *in vivo*, demonstrating that antibody-mediated targeting can redirect functional mRNA expression to a defined immune cell population.^63^ Similarly, CD117-targeted LNPs delivered mRNA to hematopoietic stem and progenitor cells and supported *in vivo* genetic modification or non-genotoxic conditioning strategies in preclinical models.^64^^,^^65^ Collectively, these studies show that extrahepatic delivery can be achieved through complementary mechanisms, including discovery-guided formulation screening, composition-dependent endogenous targeting, protein-corona engineering, rational lipid chemistry, and receptor-specific ligand targeting. Nevertheless, most of these systems remain preclinical, and organ-level accumulation does not necessarily establish productive delivery to the therapeutically relevant cell population. Extrahepatic targeting should therefore be evaluated by cell-resolved cytosolic expression and functional activity, together with off-target expression, tolerability, repeat-dose performance, and manufacturability.^55^^,^^57^^,^^63^ Local administration can be advantageous when regional expression is preferable to systemic exposure. Intratumoral mRNA delivery has produced local immunomodulatory activity in clinical studies of mRNA-2752,^36^^,^^66^ whereas aerosolized cystic fibrosis transmembrane conductance regulator (CFTR) mRNA has provided a clinical test of lung-directed delivery.^67^ Together with liver-directed systemic programs such as mRNA-3927 and NTLA-2001.^14^^,^^15^ these studies illustrate that route and tissue access are application-specific determinants of therapeutic performance. A formulation and route that are sufficient for intramuscular vaccination cannot therefore be assumed to satisfy the requirements of lung-directed protein replacement, liver-selective genome editing, or intratumoral immunomodulation.

### Stability, storage, and formulation engineering

The stability of mRNA-LNP products is governed by both RNA integrity and nanoparticle structure.^51^ The mRNA cargo is susceptible to hydrolysis and nuclease-mediated degradation, whereas the lipid components can undergo oxidation, hydrolysis, aggregation, or structural rearrangement during storage.^51^ An additional and mechanistically distinct degradation pathway arises from chemical cross-reactivity between mRNA and reactive impurities or degradation products derived from ionizable lipids. Oxidation of tertiary-amine-containing ionizable lipids can generate N-oxide intermediates that subsequently hydrolyze to electrophilic aldehydes. These reactive species covalently modify mRNA nucleobases and generate heterogeneous lipid-mRNA adducts that accumulate during storage, render the modified transcripts poorly translatable, and reduce protein expression.^68^ Importantly, these adducts do not necessarily involve RNA fragmentation and may therefore remain undetected by conventional size-based assays such as capillary electrophoresis; reversed-phase ion-pair high-performance liquid chromatography (HPLC) and mass-spectrometric nucleoside analysis are more capable of detecting the resulting changes in RNA hydrophobicity and chemical composition.^68^ Subsequent mechanistic studies have further shown that peroxide degradation within unsaturated ionizable lipid tails can generate multiple reactive aldehyde species and that adduct formation is influenced by ionizable lipid lot quality and storage temperature.^69^ These findings indicate that ionizable lipid purity, oxidative degradants, reactive aldehyde content, and lipid-mRNA adduct formation should be considered critical quality attributes rather than being subsumed under general measurements of RNA integrity. Mitigation requires stringent control of lipid raw materials and oxidative degradation pathways, together with orthogonal analytical methods and chemically stable ionizable lipid structures. For example, piperidine-based ionizable lipids limited reactive aldehyde generation, mRNA-lipid adduct formation, and loss of mRNA activity during refrigerated liquid storage.^70^ As a result, mRNA-LNP stability must be assessed at multiple levels, including RNA integrity, encapsulation efficiency, particle size, polydispersity, lipid degradation, pH, and biological potency.^51^ Cold-chain storage has been important for the initial clinical deployment of mRNA vaccines because low temperatures reduce RNA degradation and help maintain LNP stability.^51^ However, stringent cold-chain requirements increase distribution complexity, cost, and inequity, especially in regions with limited infrastructure.^51^ Accordingly, improving thermostability remains an important translational objective because less restrictive storage conditions would reduce distribution burden. Cryoprotectants and lyoprotectants, including sugars such as sucrose and trehalose, can reduce freezing- or drying-associated stress by stabilizing lipid membranes and limiting particle aggregation.^51^ Lyophilization is an attractive strategy for improving long-term storage because it can reduce water-mediated degradation pathways and potentially enable less restrictive distribution conditions.^52^ Nevertheless, lyophilized mRNA-LNP products require careful optimization of freezing rate, drying conditions, residual moisture, reconstitution behavior, and post-reconstitution potency.^51^ Formulation engineering also includes optimization of buffer composition, lipid molar ratios, particle size distribution, PEG-lipid content, and manufacturing parameters such as mixing rate and solvent exchange.^52^ These variables influence not only storage stability but also encapsulation efficiency, tissue distribution, endosomal escape, immunogenicity, and clinical tolerability.^51^ Taken together, these findings position formulation and delivery as core determinants of mRNA therapeutic performance rather than as secondary technical details.

### Alternative non-viral delivery platforms

Although LNPs remain the most clinically mature carriers for mRNA, alternative non-viral systems may offer distinct advantages in biodegradability, tissue-selective delivery, local administration, and material tunability. Polymeric carriers can be engineered by varying charge density, molecular architecture, degradation kinetics, and targeting functionality. Charge-altering releasable transporters initially form cationic complexes that protect mRNA and subsequently undergo charge-neutralizing rearrangement, promoting intracellular release of functional mRNA and protein expression *in vivo*.^71^ More recently, screening of poly(β-amino ester) libraries identified polymer nanoparticles that supported localized mRNA expression for up to 2 weeks and induced T cell activation and neutralizing responses following delivery of antigen-encoding mRNA in mice.^72^

Lipid-polymer hybrid nanoparticles are designed to combine the structural and chemical tunability of polymers with the assembly, membrane-interaction, and delivery properties of lipids. In one recent study, *1,2-*dioleoyl-*3*-trimethylammonium-propane (DOTAP)-incorporated poly(β-amino ester)-lipid hybrid nanoparticles mediated robust mRNA expression in the lungs and spleen following intravenous administration in mice, demonstrating that hybrid composition can be adjusted to support extrahepatic delivery.^73^ These systems may also permit the incorporation of degradable or stimulus-responsive polymer domains, although their increased compositional complexity creates additional challenges for formulation reproducibility and analytical characterization.

Extracellular vesicles represent a biologically derived delivery strategy that uses cell-secreted membrane vesicles to transport mRNA. Cellular nanoporation substantially increased the production and mRNA loading of engineered exosomes and enabled functional delivery of PTEN mRNA in orthotopic glioma models in mice.^74^ In a separate protein-replacement application, fibroblast-derived extracellular vesicles carrying COL1A1 mRNA produced prolonged and spatially uniform collagen expression when administered to photoaged mouse skin using a microneedle array.^75^ However, systematic comparisons of extracellular vesicle loading methods have shown that exogenous mRNA incorporation remains inefficient and is accompanied by substantial cargo loss, creating challenges for large-scale implementation and reproducible potency.^76^

These alternative systems should therefore be viewed as extensions of the mRNA delivery design space rather than as universally superior replacements for LNPs. Their translational value will depend on application-specific balances among cargo loading, tissue distribution, intracellular release, repeat-dose compatibility, manufacturing scalability, and analytical control.^71^^,^^73^^,^^74^^,^^76^

## mRNA vaccines in infectious disease and cancer immunotherapy

### Platform validation and expansion across infectious diseases

The clinical success of SARS-CoV-2 mRNA vaccines established the core translational value of the platform: once the sequence of a target pathogen is available, the encoded antigen can be redesigned rapidly while preserving the same underlying manufacturing logic and delivery framework.^2^^,^^3^ In this sense, COVID-19 converted mRNA vaccination from a promising experimental technology into a clinically validated platform by demonstrating large-scale efficacy, acceptable safety, and real-world deployability across global populations.^2^^,^^3^ Beyond COVID-19, respiratory viruses have become the most advanced follow-on indications for infectious disease mRNA vaccination. In seasonal influenza, mRNA-1010 was initially evaluated as a quadrivalent mRNA vaccine encoding influenza hemagglutinin antigens and induced strain-matched hemagglutination inhibition responses in first-in-human testing; a subsequent phase 3 trial of a trivalent formulation demonstrated superior efficacy to a licensed standard-dose influenza vaccine in adults 50 years of age or older.^77^^,^^78^ In respiratory syncytial virus (RSV), mRNA-1345 (mRESVIA), an mRNA vaccine encoding the stabilized prefusion RSV F antigen, reduced RSV-associated lower respiratory tract disease in adults 60 years of age or older and generated robust neutralizing responses in the pivotal ConquerRSV program.^79^^,^^80^ Earlier work in rabies and Zika had already shown that the platform was not limited to coronaviruses. CV7201 provided a first-in-human proof of concept for prophylactic mRNA vaccination against rabies, whereas a single low-dose nucleoside-modified mRNA-LNP vaccine protected mice and non-human primates against Zika virus challenge.^8^^,^^81^ HIV remains a particularly demanding target, but germline-targeting mRNA candidates such as eOD-GT8 60-mer have entered phase 1 clinical evaluation, illustrating how mRNA can support iterative immunogen design even when protective correlates remain unresolved (ClinicalTrials.gov: NCT05001373). Together, these studies show that the main strength of the platform is not its attachment to any single pathogen, but rather its ability to reuse a programmable expression system across rapidly changing infectious targets.

### Individualized neoantigen mRNA vaccines for precision oncology

Personalized neoantigen mRNA vaccines begin with sequencing of an individual patient’s tumor and matched normal material, followed by computational prioritization of mutation-derived epitopes and rapid manufacturing of a bespoke RNA construct encoding multiple neoantigens.^82^^,^^83^^,^^84^ Among cancer applications, this strategy most clearly demonstrates the programmability of mRNA because the platform chemistry can remain largely constant while the encoded antigenic content is rewritten for each patient.^82^^,^^83^ Clinical studies now provide proof of principle across several settings. In melanoma, personalized RNA mutanome vaccination induced poly-specific CD4+ and CD8+ T cell responses and provided the first-in-human demonstration that individualized RNA vaccines can mobilize therapeutic immunity against patient-specific mutations.^82^ In resected pancreatic ductal adenocarcinoma, adjuvant autogene cevumeran was feasible in real time after surgery, generated substantial neoantigen-specific T cells in a subset of patients, and vaccine-expanded clonotypes persisted for up to 2 years.^83^ More recently, individualized neoantigen therapy mRNA-4157 (V940) combined with pembrolizumab prolonged recurrence-free survival relative to pembrolizumab alone in resected high-risk melanoma, supporting the clinical maturation of the personalized mRNA vaccine concept.^84^ These findings are important because they suggest that individualized mRNA vaccination is no longer only immunologically plausible but increasingly operationally and clinically feasible.^82^^,^^83^^,^^84^

### Off-the-shelf mRNA vaccines targeting shared tumor antigens

Shared tumor antigen vaccines take the opposite design approach. Instead of encoding private mutations unique to a single tumor, they target recurrent tumor-associated antigens expressed across multiple patients, enabling an off-the-shelf product that is operationally simpler than fully individualized vaccination.^85^^,^^86^ This approach is attractive when rapid treatment initiation and scalable manufacturing are priorities, but it is biologically constrained by variable antigen expression across tumors, the possibility of antigen loss, and the generally lower immunogenicity of non-mutant self-derived targets compared with private neoantigens.^85^^,^^86^^,^^87^ Representative clinical studies illustrate both the promise and the limitations of this strategy. In advanced melanoma, the FixVac candidate BNT111 encoded four non-mutated melanoma-associated antigens—NY-ESO-1, MAGE-A3, tyrosinase, and TPTE—and induced strong CD4+ and CD8+ responses together with durable objective responses in checkpoint-inhibitor-experienced patients.^85^ In non-small cell lung cancer (NSCLC), BI1361849 (CV9202) encoded six NSCLC-associated antigens and showed acceptable tolerability with evidence of immune activation in a phase 1b study combined with local radiation.^87^ Together, these data suggest that shared-antigen mRNA vaccines can be clinically active but that antigen selection, tumor coverage, and resistance through antigen heterogeneity remain central determinants of success.^85^^,^^87^

### Synergy with immune checkpoint blockade

Combination with immune checkpoint blockade is a particularly rational development path for mRNA cancer vaccines because the two modalities act at complementary points in the cancer-immunity cycle.^88^ mRNA vaccination is intended to prime and expand tumor-reactive T cells, whereas PD-1/PD-L1 blockade helps preserve or restore the effector function of those T cells after they encounter an immunosuppressive tumor microenvironment.^85^^,^^88^ For this reason, the most convincing clinical signals in recent years have generally emerged from vaccine-plus-checkpoint regimens rather than from vaccine monotherapy in heavily pretreated disease.^84^^,^^85^^,^^89^ Published studies already support this framework. BNT111 showed activity as monotherapy and in combination with PD-1 blockade in checkpoint-inhibitor-treated melanoma.^85^ Autogene cevumeran elicited poly-epitopic neoantigen-specific CD4+ and/or CD8+ responses in advanced solid tumors when given alone or with atezolizumab, and the same program informed subsequent development in the adjuvant pancreatic setting.^83^^,^^89^ Most notably, mRNA-4157 (V940) plus pembrolizumab improved recurrence-free outcomes in resected high-risk melanoma, making combination-oriented development the clearest current path for translating mRNA cancer vaccines into standard oncology practice.^84^ Taken together, current evidence suggests that mRNA vaccines are most likely to succeed clinically when they are used to generate or broaden tumor-reactive T cell repertoires, while checkpoint inhibitors are used to sustain the function of those repertoires *in vivo*.^83^^,^^84^^,^^85^^,^^89^ Overall, infectious disease and oncology studies together show that the key advantage of mRNA vaccination is not limited to speed alone but lies in a programmable platform that can be rapidly redirected toward viral antigens, patient-specific neoantigens, or shared tumor-associated targets depending on clinical need.^2^^,^^3^^,^^82^^,^^83^^,^^84^^,^^85^

### Therapeutic applications beyond vaccination

Clinical development beyond vaccination has clarified that mRNA is not inherently a vaccine modality but a transient protein-expression platform that can be redirected toward multiple therapeutic goals. In this broader setting, the encoded sequence is no longer limited to an antigen for immune priming but may instead specify a deficient enzyme, a functional membrane protein, a secreted growth factor, a monoclonal antibody, an immunomodulator, or a genome-editing enzyme. Accordingly, the major translational variables shift from antigen selection alone to tissue accessibility, required expression kinetics, repeat-dosing feasibility, and therapeutic index. These beyond-vaccine applications differ not only in the encoded payload but also in the biological constraints that determine clinical feasibility. Enzyme replacement, pulmonary gene complementation, regenerative signaling, *in vivo* antibody production, intratumoral immunomodulation, genome editing, and transient cell engineering each impose different requirements for target tissue access, route of administration, expression duration, repeat dosing, and tolerability. Figure 2 provides an application-level map of these use cases, highlighting how mRNA can be redirected across therapeutic contexts by changing the encoded sequence, delivery strategy, and desired expression kinetics.

**Figure 2:**
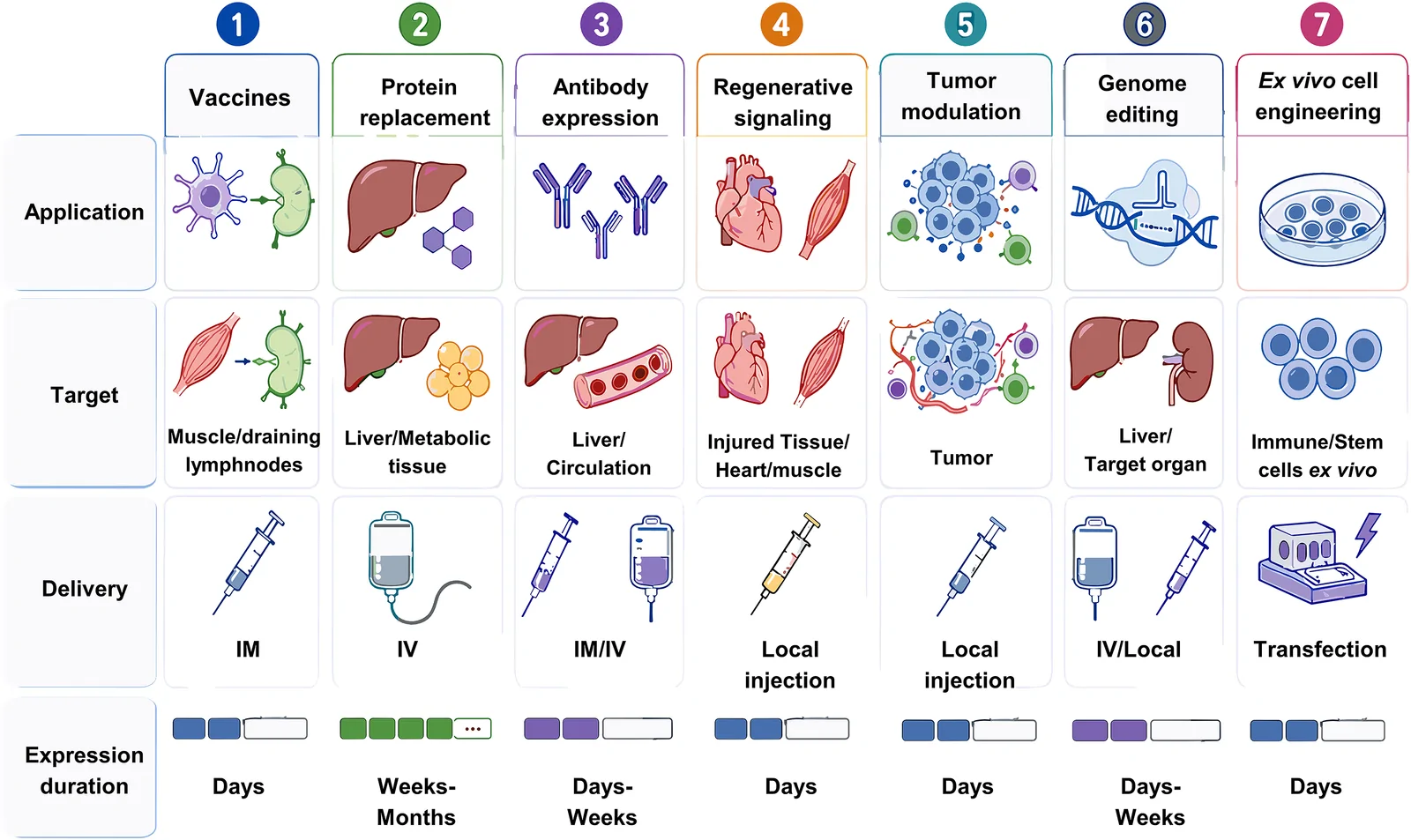
Application map of mRNA medicine beyond vaccines The schematic compares major therapeutic applications according to encoded payload, target tissue, delivery route, and expected expression duration. This schematic figure was prepared based on author-provided conceptual sketches and scientific layout, using FigureLab for scientific illustration and manual author editing.

The translational value of these applications cannot be judged solely by whether the encoded protein can be produced. Instead, each therapeutic setting represents a different stress test of the mRNA platform. Critical variables include whether the target tissue is accessible to current delivery systems, whether the therapeutic protein requires intracellular assembly or secretion, whether transient expression is sufficient or repeated dosing is unavoidable, and whether excessive or off-target expression carries reversible or irreversible consequences. These differences help explain why liver-directed enzyme replacement has generated encouraging clinical signals, whereas pulmonary protein replacement remains constrained by delivery despite a strong molecular rationale, and why transient mRNA exposure can be particularly advantageous when it produces a longer-lived secreted biologic or a permanent genomic edit.^13^^,^^14^^,^^15^^,^^67^

### *In vivo* protein replacement through transient enzyme expression

One of the clearest non-vaccine applications of mRNA is protein replacement therapy, in which the transcript is designed to restore expression of a missing or dysfunctional enzyme rather than encode an immunogen. This strategy is especially attractive for liver-directed inborn errors of metabolism because intravenously administered LNPs naturally favor hepatic delivery, allowing transient translation of the therapeutic protein in the organ in which metabolic correction is required. The strongest current clinical example is mRNA-3927 for propionic acidaemia, a dual mRNA therapy encoding the propionyl-CoA carboxylase alpha subunit (PCCA) and propionyl-CoA carboxylase beta subunit (PCCB) subunits of propionyl-CoA carboxylase. In the first-in-human phase 1/2 study, no dose-limiting toxicities were reported, and interim analyses suggested a 70% reduction in the risk of metabolic decompensation events among participants who had experienced such events during the pretreatment period.^15^ mRNA-3927 is mechanistically informative because successful treatment requires more than expression of a single soluble protein. Functional propionyl-CoA carboxylase is a multimeric mitochondrial enzyme requiring coordinated production of both PCCA and PCCB subunits, appropriate subunit stoichiometry, and mitochondrial localization. In preclinical studies, co-delivery of the two mRNAs restored hepatic PCC activity and reduced disease-associated metabolites during repeated dosing, whereas delivery of either transcript alone was insufficient for comparable enzyme restoration.^90^ The clinical study further provides an unusually relevant test of chronic mRNA administration: 16 participants received a total of 346 intravenous doses, with no dose-limiting toxicities observed.^15^ However, the reported reduction in metabolic decompensation events was derived from an interim, uncontrolled analysis of the subgroup with events during the pretreatment period and should therefore be interpreted as an encouraging pharmacologic and clinical signal rather than definitive efficacy.^15^ This program illustrates why liver-directed metabolic diseases are currently among the most tractable settings for non-vaccine mRNA: hepatic delivery is comparatively efficient, biochemical pharmacodynamic markers are measurable, and intermittent restoration of enzyme activity may be clinically meaningful despite transient expression.^15^^,^^90^

### Lung-directed CFTR restoration as a delivery stress test

Cystic fibrosis highlights both the promise and the difficulty of pulmonary mRNA therapy. In principle, delivery of CFTR mRNA to airway epithelial cells could provide a genotype-agnostic strategy for restoring CFTR protein function, making mRNA attractive even in settings where mutation-specific small molecules are insufficient or not applicable. In practice, however, airway mucus, epithelial access, regional deposition, and repeat-dose tolerability make the lung one of the most demanding delivery targets in mRNA medicine. MRT5005, an aerosolized LNP formulation containing codon-optimized CFTR mRNA, provided a first-in-human test of this concept. In the phase 1/2 study, treatment was generally safe and well tolerated overall, but febrile and hypersensitivity reactions occurred in some participants, and no consistent improvement in FEV1 was observed, illustrating that pulmonary delivery remains a major translational bottleneck even when the therapeutic rationale is strong.^67^ MRT5005 also illustrates why anatomical access to an organ should not be equated with productive delivery to the relevant cell population. In the phase 1/2 study, 31 participants received MRT5005 across single-, weekly multiple-, or daily-dose regimens; febrile reactions occurred in 10 treated participants, two hypersensitivity reactions were reported, and no consistent improvement in FEV1 was observed.^67^ Thus, direct aerosol administration alone was insufficient to overcome the combined barriers of mucus, regional deposition, epithelial uptake, endosomal escape, and the level of CFTR expression required for physiological correction. Importantly, this clinical result does not establish that CFTR mRNA replacement is intrinsically ineffective. A more recent LUNAR-CFTR formulation restored CFTR-mediated ion transport in human bronchial epithelial cultures and improved mucociliary clearance approximately 3-fold after a single dose in cystic fibrosis (CF) ferrets, although these findings remain preclinical.^91^ The contrast between MRT5005 and newer pulmonary formulations therefore supports a formulation-specific interpretation of clinical failure and identifies productive airway epithelial delivery, rather than CFTR coding capacity itself, as the principal development bottleneck.^67^^,^^91^

### Transient growth factor expression for regenerative repair

Beyond replacement of missing proteins, mRNA can also be used to generate a short-lived burst of a therapeutic factor intended to reshape tissue repair. This category is conceptually distinct from classical enzyme replacement because the goal is not the permanent restoration of endogenous protein deficiency but the transient induction of a regenerative or pro-angiogenic program. AZD8601, an mRNA encoding VEGF-A, is the leading clinical example of this approach. In the EPICCURE study, naked VEGF-A mRNA was delivered via direct intramyocardial injection during coronary artery bypass grafting, demonstrating that local and transient therapeutic protein expression can be pursued without systemic LNP administration. The study was small and underpowered for efficacy, but it established feasibility and an encouraging safety profile, with no treatment-related serious adverse events and exploratory signals consistent with possible biologic activity.^92^ The AZD8601 program also illustrates a setting in which transient expression may be a design advantage rather than a limitation. In an earlier first-in-human study in men with type 2 diabetes, intradermal administration produced detectable local VEGF-A for approximately 4–24 h and increased local skin blood flow without requiring a nanoparticle carrier.^93^ EPICCURE subsequently extended this local expression strategy to the myocardium, but only seven patients received AZD8601 and four received placebo, precluding meaningful efficacy conclusions despite favorable safety observations.^92^ These studies suggest that a short, spatially confined pulse of a regenerative factor may avoid the need for prolonged systemic exposure; however, the requirement for direct tissue injection also limits scalability and restricts this strategy to anatomically accessible or procedurally exposed tissues.^92^^,^^93^

### mRNA-encoded antibodies as *in vivo* biologics

mRNA-encoded antibodies extend the same logic from endogenous proteins to recombinant biologics. Instead of manufacturing a monoclonal antibody *ex vivo* and infusing the purified protein, this strategy delivers mRNA encoding the antibody chains so that host cells transiently produce the biologic *in vivo*. This concept is attractive because it can, in principle, simplify the production of complex proteins and turn the body into a temporary bioreactor. The best human proof of concept to date is mRNA-1944, which encodes the heavy and light chains of the anti-chikungunya monoclonal antibody CHKV-24. In a phase 1 trial, mRNA-1944 demonstrated *in vivo* antibody expression together with detectable *ex vivo* neutralizing activity, providing the first clinical evidence that an intravenously delivered mRNA therapeutic can produce a functional monoclonal antibody in humans.^13^ The pharmacologic logic of mRNA-encoded antibodies differs from conventional protein replacement because the lifetime of the therapeutic effect is not determined solely by the lifetime of the mRNA. In the mRNA-1944 trial, single intravenous doses produced dose-dependent CHKV-24 antibody concentrations, and therapeutically relevant antibody levels persisted for at least 16 weeks at the 0.3 and 0.6 mg/kg dose levels; a second 0.3 mg/kg dose increased antibody exposure by approximately 1.8-fold.^13^ This prolonged pharmacodynamic effect reflects continued exposure to the secreted antibody after the encoding transcript has declined and illustrates how a transient RNA payload can generate a substantially longer-lived protein product.^13^ At the same time, this creates a two-stage pharmacokinetic problem in which mRNA delivery and translation determine the rate of antibody production, whereas secretion, tissue distribution, and IgG clearance determine systemic exposure. Control of heavy- and light-chain expression, peak antibody concentration, and interindividual variability therefore becomes as important as mRNA transfection efficiency itself. Moreover, mRNA-1944 established pharmacologic proof of concept in healthy volunteers rather than therapeutic efficacy in infected patients, and the clinical value of this strategy remains to be demonstrated in disease-specific trials.^13^

### Local tumor remodeling with mRNA-encoded immunomodulators

In oncology, mRNA can also be used outside the conventional vaccine framework by encoding cytokines, costimulatory ligands, or other immune agonists intended to remodel the tumor microenvironment locally. The conceptual advantage of this approach is that it seeks to concentrate immune activation within the tumor rather than rely on systemic cytokine administration, which has historically been limited by toxicity. Preclinical studies showed that intratumoral delivery of mRNAs encoding IL-23 and IL-36γ, as well as local mRNA combinations containing OX40L and other costimulatory ligands, can provoke robust local inflammation and systemic antitumor immunity.^94^^,^^95^ Clinically, mRNA-2752, which encodes OX40L, IL-23, and IL-36γ, has provided a representative translational example. In a phase 1 study of high-risk ductal carcinoma *in situ*, intratumoral mRNA-2752 combined with pembrolizumab appeared safe and was associated with rapid regression in immune-infiltrated lesions, supporting the broader concept of local mRNA-based cancer immunomodulation.^66^ More mature clinical evidence has now emerged from a phase 1 study of intratumoral mRNA-2752 alone or in combination with durvalumab in 134 patients with advanced solid tumors.^36^ The maximum tolerated dose was not reached, although two grade 2 cytokine-release syndrome events occurred in the combination arm, and grade 3 mRNA-2752-related adverse events were reported in 25.2% of patients receiving combination therapy.^36^ In the checkpoint-inhibitor-resistant melanoma cohort, the confirmed objective response rate was 17.9%, and the disease-control rate was 42.9%, with pharmacodynamic analyses showing sustained inflammatory remodeling of the tumor microenvironment, particularly in responding patients.^36^ These findings strengthen the mechanistic rationale for using mRNA to generate potent immunomodulators locally, but they also expose the principal limitation of this strategy: direct intratumoral dosing requires accessible lesions and does not by itself solve delivery to disseminated or anatomically inaccessible disease. Local expression may therefore improve the therapeutic index of inflammatory payloads, but clinical scalability depends strongly on tumor accessibility and the ability of local immune activation to generate systemic antitumor effects.^36^^,^^66^^,^^94^

### Transient delivery of genome-editing machinery

Genome editing represents a mechanistically distinct application in which mRNA serves as a transient delivery format for an editing enzyme rather than as the therapeutic product itself. This feature is particularly attractive because editor exposure can be short-lived, while the resulting genomic modification is permanent. The first-in-human NTLA-2001 program, now termed nexiguran ziclumeran, established the clinical feasibility of this strategy by delivering Cas9 mRNA together with a transthyretin (TTR)-targeting single guide RNA in a liver-directed LNP.^14^ Subsequent clinical follow-up has demonstrated the durability of this single-dose approach. In patients with transthyretin amyloidosis (ATTR) cardiomyopathy, one intravenous infusion reduced serum TTR by approximately 89% at day 28 and 90% at 12 months.^96^ Similarly, in hereditary ATTR with polyneuropathy, serum TTR was reduced by approximately 90% at day 28 and remained reduced by 92% at month 24 after a single administration.^97^ These findings illustrate an important inversion of the conventional mRNA paradigm: short RNA persistence is advantageous when a transient pulse of editor expression is sufficient to generate a durable therapeutic effect.

The same logic underlies PCSK9-directed base editing. VERVE-101 packages base-editor mRNA and guide RNA in an LNP designed for hepatocyte delivery, and preclinical studies in non-human primates demonstrated durable PCSK9 editing and LDL-cholesterol reduction after a single intravenous administration.^98^^,^^99^^,^^100^ However, the permanence of genome editing also changes the dominant safety requirements. Whereas repeated administration and limited expression duration constrain conventional protein-replacement mRNA, genome-editing applications place greater emphasis on tissue-selective delivery, editing specificity, and long-term assessment of unintended or irreversible genomic changes. Thus, the therapeutic value of mRNA in genome editing derives not from prolonged RNA expression but from efficient delivery of a short, spatially controlled editor pulse capable of generating a durable biological effect.^14^^,^^96^^,^^97^^,^^98^

### Transient mRNA engineering of immune cells

*Ex vivo* immune cell engineering represents another setting in which the transient nature of mRNA can be exploited deliberately rather than regarded as a limitation. Electroporation of receptor-encoding mRNA enables temporary CAR or TCR expression without genomic integration, allowing engineered cell activity to decline as the introduced transcript is degraded.^101^ This reversibility can be particularly valuable when sustained receptor expression creates a risk of on-target, off-tumor toxicity. In hepatitis B virus (HBV)-related hepatocellular carcinoma, for example, HBV antigens can be expressed by both malignant cells and non-malignant HBV-infected hepatocytes. HBV-specific T cells were therefore engineered using TCR-encoding mRNA so that receptor activity would remain transient and escalating doses could be administered while limiting the duration of potential hepatotoxicity.^102^

The corresponding trade-off is reduced cellular persistence and a greater requirement for repeated manufacturing and administration. In a phase 1 study of patients with recurrent HBV-related hepatocellular carcinoma after liver transplantation, six patients received a median of 6.5 infusions of mRNA-electroporated HBV-specific TCR T cells; treatment was generally well tolerated, with no cytokine-release syndrome or neurotoxicity reported.^103^ A separate phase 1 study of intravenously administered RNA-electroporated mesenchymal–epithelial transition factor (cMET)-directed CAR T cells in metastatic melanoma and breast carcinoma similarly demonstrated feasibility and an acceptable safety profile, although antitumor activity was limited and CAR T cell signals were not detected in post-treatment tumor biopsies.^104^ The Radium-1 program has provided an additional first-in-human example in which autologous T cells transiently expressing an mRNA-encoded TCR were administered repeatedly to a patient with treatment-refractory microsatellite instability (MSI)-high colorectal cancer, demonstrating feasibility while maintaining reversibility of receptor expression.^105^ Collectively, these studies show that transient mRNA cell engineering is most attractive when controllability and reversibility are prioritized over long-term receptor persistence; conversely, indications requiring sustained cellular activity may favor permanently engineered cell products.

Clinical maturity remains uneven across the mRNA field. Currently approved products are predominantly prophylactic vaccines, whereas cancer immunotherapies and mRNA-enabled genome-editing medicines have only recently entered phase 2/3 or phase 3 development. Selected approved and late-stage clinical programs are summarized in Table 1. By pairing each product code with its encoded payload, molecular target, indication, delivery route, and development status, the table illustrates that clinical maturity depends not simply on the ability of mRNA to express a functional protein but on the compatibility of the RNA platform and delivery strategy with the biological requirements of each indication.

**Table 1:** Selected approved and late-stage clinical mRNA medicines

Product and platform	Encoded payload or molecular target	Indication	Delivery format and route	Regulatory or clinical status	Key evidence or trial
COMIRNATY (BNT162b2; conventional mRNA)	prefusion-stabilized SARS-CoV-2 spike antigen	prevention of COVID-19	LNP; intramuscular	approved, including in the United States	Polack et al.^2^
SPIKEVAX (mRNA-1273; conventional mRNA)	prefusion-stabilized SARS-CoV-2 spike antigen	prevention of COVID-19	LNP; intramuscular	approved, including in the United States	Baden et al.^3^
MRESVIA (mRNA-1345; conventional mRNA)	prefusion-stabilized RSV F antigen	prevention of RSV-associated lower respiratory tract disease	LNP; intramuscular	approved in the United States	Wilson et al.^79^
MNEXSPIKE (mRNA-1283; conventional mRNA)	SARS-CoV-2 spike receptor-binding and N-terminal domains	prevention of COVID-19	LNP; intramuscular	approved in the United States	Chalkias et al.^106^
Kostaive (zapomeran; ARCT-154; self-amplifying RNA)	self-amplifying RNA encoding SARS-CoV-2 spike and replicase machinery	prevention of COVID-19	LNP; intramuscular	approved in the European Union and other jurisdictions	Oda et al.^107^
MFLUSIVA (mRNA-1010; conventional mRNA)	hemagglutinin antigens from seasonal influenza strains	prevention of influenza in persons ≥50 years of age	LNP; intramuscular	approved in the United States	Leroux-Roels et al.^78^; ClinicalTrials: NCT06602024
mCOMBRIAX (mRNA-1083; multicomponent conventional mRNA)	seasonal influenza and SARS-CoV-2 antigens	prevention of influenza and COVID-19 in persons ≥50 years of age	LNP; intramuscular	approved in the European Union	Rudman et al.^108^; ClinicalTrials.gov: NCT06097273
mRNA-1403 (multivalent conventional mRNA)	antigens representing clinically relevant norovirus genotypes	prevention of norovirus acute gastroenteritis	LNP; intramuscular	phase 3, active and not recruiting	ClinicalTrials.gov: NCT06592794
Intismeran autogene (V940/mRNA-4157; individualized mRNA)	patient-specific tumor neoantigens	adjuvant treatment of resected high-risk melanoma in combination with pembrolizumab	LNP; intramuscular	phase 3, active and not recruiting	Weber et al.^84^; ClinicalTrials.gov: NCT05933577
BNT113 (off-the-shelf mRNA cancer immunotherapy)	HPV16 E6 and E7 oncoproteins	unresectable recurrent or metastatic HPV16-positive head and neck squamous cell carcinoma	RNA-lipoplex; intravenous	phase 2/3, recruiting	ClinicalTrials.gov: NCT04534205
Nexiguran ziclumeran (NTLA-2001; mRNA-enabled genome editing)	Cas9 mRNA and a TTR-targeting single guide RNA	transthyretin amyloidosis with cardiomyopathy or hereditary polyneuropathy	liver-directed LNP; intravenous	phase 3, recruiting	Gillmore et al.^14^; ClinicalTrials.gov: NCT06128629 and NCT06672237

Approved indicates regulatory authorization in at least one specified jurisdiction. Late-stage development was defined as an active or completed phase 2/3 or phase 3 program. Regulatory and clinical development status was current as of August 2026. COVID-19, coronavirus disease 2019; HNSCC, head and neck squamous cell carcinoma; HPV16, human papillomavirus type 16; LNP, lipid nanoparticle; RSV, respiratory syncytial virus; TTR, transthyretin.

Taken together, these therapeutic programs demonstrate that transient expression is neither uniformly advantageous nor uniformly limiting; its value depends on the biological objective of each application. In chronic enzyme replacement, transient expression necessitates repeated systemic administration but may remain clinically acceptable when the target organ is readily accessible and biochemical correction can be monitored, as illustrated by liver-directed mRNA-3927.^15^ In pulmonary CFTR replacement, by contrast, the major limitation is productive delivery to the appropriate airway epithelial population rather than the ability of mRNA to encode functional CFTR.^67^^,^^91^ Regenerative applications can exploit a short and spatially restricted burst of growth factor expression, whereas mRNA-encoded antibodies illustrate that a transient RNA template can generate a protein product with substantially longer systemic persistence.^13^^,^^92^^,^^93^ Intratumoral immunomodulation similarly benefits from localized and temporary expression of inflammatory mediators but remains constrained by lesion accessibility.^36^^,^^66^ Genome editing represents the opposite pharmacologic extreme: transient editor expression is desirable precisely because the resulting genomic modification is durable, shifting the principal concern from repeat dosing toward delivery specificity and irreversible genomic consequences.^14^^,^^96^^,^^97^ In *ex vivo* cell engineering, transient receptor expression increases reversibility and controllability at the cost of limited persistence and repeated cell product administration.^102^^,^^103^^,^^104^

These contrasts define the central translational framework of this review. Therapeutic success cannot be achieved simply by exchanging the coding sequence within an otherwise interchangeable mRNA platform. Instead, each application requires alignment among RNA architecture, delivery system, administration route, target cell accessibility, required expression magnitude and duration, acceptable off-target exposure, and repeat-dose burden. The most tractable applications are therefore those in which current delivery systems efficiently access the relevant tissue, local administration is feasible, or transient expression itself provides a defined pharmacologic or safety advantage. Conversely, applications requiring sustained high-efficiency delivery to difficult extrahepatic cell populations remain substantially more demanding. These application-specific differences in payload, delivery route, target tissue, expression kinetics, repeat-dose requirements, and dominant translational bottlenecks are summarized in Table 2.

**Table 2:** Application matrix of mRNA medicines by therapeutic objective, delivery route, target tissue, and dominant translational constraint

Therapeutic use	Payload class	Route/target site	Repeat-dose profile	Key bottleneck and examples
Prophylactic vaccination	pathogen antigen	i.m.; local cells, APCs, draining LNs	high; booster or seasonal dosing feasible	reactogenicity, variant matching, stability, scale-up; COMIRNATY, SPIKEVAX, mRESVIA, mRNA-1010, ARCT-154
Therapeutic cancer vaccination	neoantigens or shared tumor antigens	i.m./s.c.; APCs and lymphoid tissues	moderate; repeated priming feasible	neoantigen selection, tumor heterogeneity, TME suppression, manufacturing turnaround; V940/mRNA-4157, autogene cevumeran, BNT111, CV9202
Protein replacement	enzyme, membrane protein, or secreted protein	i.v. LNP or local delivery; liver, lung, or disease tissue	moderate; chronic dosing often required	organ-specific delivery, expression magnitude, repeat-dose tolerability; mRNA-3927, MRT5005
*In vivo* antibody production	antibody heavy/light chains	i.v. LNP; liver or secretory tissues	moderate; limited by exposure control and immunogenicity	PK control, chain balance, anti-drug response; mRNA-1944
Local regenerative signaling	growth factor or repair protein	local injection; injured or ischemic tissue	moderate to limited	spatial confinement, ectopic expression, functional benefit; AZD8601
Local tumor immunomodulation	cytokines, costimulatory ligands, immune agonists	intratumoral; TME and immune infiltrates	moderate if lesions are accessible	local retention, cytokine toxicity, systemic antitumor immunity; mRNA-2752
*In vivo* genome editing	Cas9/base-editor mRNA plus guide RNA	i.v. LNP; mainly hepatocytes	limited; usually one-time or infrequent dosing	tissue-selective delivery, off-target editing, editor immunity, long-term safety; NTLA-2001, VERVE-101
*Ex vivo* cell engineering	CAR, TCR, or editor mRNA	*ex vivo* electroporation; T cells or NK cells	high at cell-product level	transient expression, cell fitness, manufacturing burden; cMET CAR-T, HBV-TCR, Radium-1 TCR cells

APC, antigen-presenting cell; CAR, chimeric antigen receptor; i.m., intramuscular; i.v., intravenous; LN, lymph node; LNP, lipid nanoparticle; PK, pharmacokinetic; s.c., subcutaneous; TCR, T cell receptor; TME, tumor microenvironment.

## Next-generation mRNA platforms

### saRNA

saRNA is designed to preserve the core message-encoding logic of conventional mRNA while adding replicase machinery that allows intracellular RNA amplification after delivery.^109^^,^^110^ In practice, the structural genes of an alphavirus-derived replicon are replaced with the antigen or therapeutic payload, so the delivered RNA can produce both the encoded product and the replication complex needed to amplify template copies inside the cell.^109^ The major advantage of saRNA is dose efficiency. Because the transcript can self-amplify after entry into the cytoplasm, saRNA often achieves meaningful antigen expression at lower administered doses than conventional mRNA.^109^^,^^110^ Clinical development has also matured substantially: first-in-human studies such as COVAC1 showed feasibility, and later, ARCT-154 (zapomeran/Kostaive), an saRNA vaccine encoding SARS-CoV-2 spike, demonstrated that the platform can reach large efficacy trials and durable booster immunogenicity in humans.^107^^,^^110^^,^^111^ Its liabilities are equally important. saRNA constructs are substantially longer than conventional mRNAs, which complicates *in vitro* transcription (IVT), purification, and formulation; they also retain a strong tendency toward innate immune sensing, which can suppress translation if not carefully managed.^109^^,^^110^ Chemical modification of saRNA, however, introduces an additional constraint that is not encountered to the same extent with conventional mRNA. For conventional mRNA, a modified nucleoside must remain compatible with IVT, transcript stability, and ribosomal translation; for saRNA, the modified input RNA must additionally support translation of the viral replicase and remain a competent template for RNA-dependent RNA polymerase-mediated amplification. Consequently, modification chemistries that improve conventional mRNA cannot be assumed to transfer directly to saRNA. m1Ψ, for example, has shown strongly backbone-dependent effects: it impaired RNA-dependent RNA polymerase recognition and replication in a nodavirus-derived replicon, severely reduced translation in a coxsackievirus B3 replicon, and provided no clear *in vivo* advantage in a Semliki Forest virus-derived system.^112^ In a Venezuelan equine encephalitis virus-derived saRNA platform, complete substitution with m1Ψ produced no detectable reporter expression, whereas 5-methylcytidine (m5C) and 5-methyluridine (m5U) remained compatible with functional saRNA expression *in vitro* and supported sustained expression *in vivo*.^113^ Complementary systematic screening further identified 5-hydroxymethylcytidine, m5C, and m5U as fully substitutable chemistries that retained saRNA functionality while reducing interferon responses and supporting prolonged protein expression.^114^ These findings demonstrate that chemical modification of saRNA is a nucleotide- and replicon-specific optimization problem: an effective modification must reduce innate immune recognition without disrupting replicase production, template recognition, or intracellular RNA amplification. Thus, nucleoside chemistries validated for conventional mRNA require direct replication-competence testing before they can be transferred to saRNA platforms.^112^^,^^113^^,^^114^

A second challenge becomes particularly important when saRNA is considered for therapeutic applications beyond vaccination. RNA-dependent RNA polymerase-mediated amplification generates dsRNA replication intermediates, which can activate cytosolic RNA-sensing pathways including RIG-I, MDA5, PKR, and OAS/RNase L.^115^^,^^116^ Activation of these pathways induces type I and III interferon and inflammatory signaling, while PKR-mediated eIF2α phosphorylation suppresses translation and OAS/RNase L activation promotes RNA degradation. Experimental suppression of these responses with viral immune-evasion proteins markedly increased saRNA-encoded protein expression, directly demonstrating that replication-associated innate sensing can limit productive expression.^115^^,^^116^ This property has different implications for vaccines and non-vaccine therapeutics. In vaccination, replication-associated innate sensing can contribute to the self-adjuvanting activity of saRNA, whereas applications requiring sustained production of a therapeutic protein may instead be limited by inflammatory cytokine production, translational shutdown, RNA degradation, and cytotoxicity. Consistent with this distinction, engineered saRNA variants with reduced activation of dsRNA-sensing pathways showed decreased interferon responses, less translational inhibition and apoptosis, and increased payload expression.^117^ More recently, immune-evasive saRNA designed specifically for durable therapeutic gene expression incorporated inhibitors of PKR, OAS/RNase L, and NF-κB signaling and achieved sustained transgene expression with reduced cytotoxicity and antiviral cytokine secretion in primary cells.^118^ Thus, for non-vaccine applications, saRNA optimization must balance the expression benefit of intracellular amplification against the innate-immunogenic cost of the replication process itself; simply increasing replication is not necessarily advantageous if the accompanying dsRNA burden suppresses productive protein expression or narrows the therapeutic window.^115^^,^^117^^,^^118^

### circRNA

circRNA is a distinct next-generation strategy in which the transcript is covalently closed rather than linear, thereby eliminating free 5′ and 3′ ends that are susceptible to exonuclease attack.^119^^,^^120^ This architecture has made circRNA attractive as a platform for more sustained protein expression, especially in applications where a longer expression window may be advantageous.^119^ At the same time, circRNA presents its own technical challenges.

Because circRNA lacks a free 5′ end and the canonical cap-poly(A) organization of linear mRNA, translation has traditionally relied on cap-independent initiation elements, particularly internal ribosome entry sites (IRESs). However, IRES activity is highly dependent on sequence, RNA structure, and cellular context rather than being an interchangeable module. High-throughput screening has identified thousands of sequences capable of supporting circRNA translation and showed that local RNA structure and complementarity to 18S rRNA contribute to IRES activity.^121^ More application-oriented optimization has further demonstrated that IRES selection can substantially alter therapeutic circRNA output. Screening of 29 IRES elements for a circRNA cancer vaccine platform identified an enterovirus A-derived IRES with strong activity in immune cells, and subsequent SHAPE-MaP-guided structural modification and spacer optimization further increased translation.^122^ More recently, a dual-IRES strategy combining an upstream encephalomyocarditis virus (EMCV)-derived IRES with a second IRES positioned downstream of the coding sequence increased reporter expression relative to corresponding single-IRES constructs, indicating that IRES architecture and positional context can be engineered rather than treated as fixed components.^123^

A distinct strategy introduces cap-dependent translation into the circular topology as an alternative to conventional IRES-mediated initiation. Chemotopological approaches have enabled covalent incorporation of an internal N7-methylguanosine (m7G) cap within circRNA, allowing recruitment of the eIF4E-eIF4G translation initiation machinery and supporting IRES-independent protein production.^124^ Related work has further shown that both covalently attached internal m7G caps and non-covalent recruitment of m7G-capped oligonucleotides can enhance circRNA translation, indicating that cap-dependent initiation represents an additional engineerable strategy for improving protein output.^125^ These findings indicate that translational efficiency is not an intrinsic consequence of RNA circularization alone but is determined by initiation mechanism, IRES architecture, cap topology, and sequence context. Accordingly, future therapeutic circRNA design will require optimization of translation initiation alongside circularization efficiency, RNA purity, stability, and immunogenicity.^121^^,^^122^^,^^123^^,^^124^^,^^125^

In addition, purification of true circRNA away from linear precursors and byproducts remains a major development issue, and immunogenicity can vary depending on the circularization chemistry, purification workflow, RNA structure, and sequence design.^120^^,^^126^ The field has nevertheless advanced rapidly. Foundational work demonstrated potent and durable protein production from engineered circRNAs,^119^ and more recent studies have shown that scarless circularization systems can improve protein output while maintaining minimal immunogenicity, which moves circRNA closer to practical therapeutic development.^120^ Even so, circRNA remains less clinically mature than conventional mRNA and, at present, should be viewed as a promising but still emerging platform rather than a clinically dominant successor.^119^^,^^120^

### Comparative perspective across mRNA platforms

Conventional mRNA, saRNA, and circRNA represent distinct solutions to the same central challenge in RNA medicine: how to achieve sufficient protein expression while balancing manufacturability, expression duration, innate immune activation, and clinical safety.^109^^,^^110^^,^^111^^,^^119^^,^^120^ Conventional mRNA is currently the most clinically validated format because its molecular architecture, manufacturing workflow, and delivery requirements are comparatively well established, but its transient expression profile may limit applications that require prolonged protein production.^109^^,^^110^ In contrast, saRNA can increase intracellular RNA abundance through replicase-mediated amplification, potentially enabling lower doses and longer antigen expression, although this benefit is offset by increased RNA length, formulation complexity, and stronger innate immune sensing.^110^^,^^119^ circRNA offers a different strategy by using a covalently closed structure that can improve resistance to exonuclease-mediated degradation and support sustained translation, but efficient translation initiation, purification, immunogenicity control, and scalable manufacturing remain important unresolved challenges.^111^^,^^120^ These differences indicate that no single mRNA platform is universally optimal. Instead, platform selection should be guided by the intended clinical use: conventional mRNA is well suited for established vaccine and short-term protein-expression applications, saRNA may be advantageous when dose sparing or prolonged antigen expression is desirable, and circRNA may be most relevant for future applications requiring extended expression from a stable RNA template.^109^^,^^110^^,^^111^^,^^119^^,^^120^ The major advantages, limitations, and suitable applications of these three platforms are summarized in Table 3.

**Table 3:** Comparative features of therapeutic RNA platform architectures

Platform	RNA architecture, expression window, and dose logic	Major immune-related issues	Manufacturing and scale-up issues	Clinical maturity and representative examples
Conventional mRNA	architecture: linear IVT RNA with 5′ cap, 5′ UTR, ORF, 3′ UTR, and poly(A) tail; often nucleoside modified and LNP formulated expression: begins within hours, peaks at ∼24–48 h, and declines over days to ∼1 week, depending on route, formulation, UTRs, and tissue dose: reference platform; generally requires higher input RNA than saRNA for comparable output	innate sensing may arise from unmodified uridine, dsRNA impurities, RNA degradation products, or LNP reactogenicity; repeat dosing may involve anti-PEG responses or accelerated blood clearance	requires control of capping, poly(A), transcript integrity, dsRNA removal, residual DNA, encapsulation, particle size, and storage stability.	maturity: most mature; multiple approved products and population-scale clinical use examples: COMIRNATY; SPIKEVAX; mRNA-1345/mRESVIA; MNEXSPIKE; V940/mRNA-4157.
Self-amplifying RNA	architecture: larger linear RNA encoding the payload and alphavirus-derived replicase machinery for intracellular amplification expression: longer than conventional mRNA; expression may persist for several days to ≥1–2 weeks, depending on construct, dose, and formulation dose: dose-sparing platform; intracellular amplification can increase output from lower input RNA	replicase activity and replication intermediates can enhance interferon signaling; excessive innate sensing may suppress translation and reduce tolerability	large RNA size complicates IVT yield, integrity, purification, formulation, encapsulation, and analytics	maturity: increasing maturity; clinical vaccine programs and regional authorization or approval examples: ARCT-154/Kostaive; COVAC1; other saRNA vaccine programs.
Circular RNA	architecture: covalently closed RNA lacking free 5′/3′ ends; translation commonly uses IRES-mediated initiation, with emerging internal cap-initiated strategies expression: potentially more sustained than linear mRNA due to exonuclease resistance, but human benchmarks remain limited dose: potential dose-sparing may be possible, but clinical dose standards remain undefined	immunogenicity depends on circularization chemistry, RNA structure, sequence design, purification, and residual linear or nicked RNA species	key barriers include circularization efficiency, byproduct removal, topology confirmation, scalable purification, potency assays, and reproducible translation	maturity: emerging; less clinically mature than conventional mRNA or saRNA examples: engineered circRNA systems; scarless circRNA platforms; early therapeutic and vaccine programs.

dsRNA, double-stranded RNA; IRES, internal ribosome entry site; IVT, *in vitro* transcription; LNP, lipid nanoparticle; mRNA, messenger RNA; ORF, open reading frame; PEG, polyethylene glycol; saRNA, self-amplifying RNA; UTR, untranslated region.

### Translational challenges and future perspectives

Across the primary mechanistic, preclinical, and clinical evidence reviewed above, four recurring constraints emerge as major determinants of translational performance: tissue-selective delivery, productive cytosolic release, immune compatibility during repeat dosing, and manufacturing reproducibility. These constraints are interconnected: tissue-selective delivery determines where the RNA goes, endosomal escape determines how much internalized RNA becomes productively available in the cytosol, innate and carrier-directed immune responses shape repeat-dose feasibility, and manufacturing quality determines whether these properties can be reproduced at clinical scale. The following discussion is therefore organized around these four constraints and their interaction across therapeutic applications. Figure 3 summarizes this framework.

**Figure 3:**
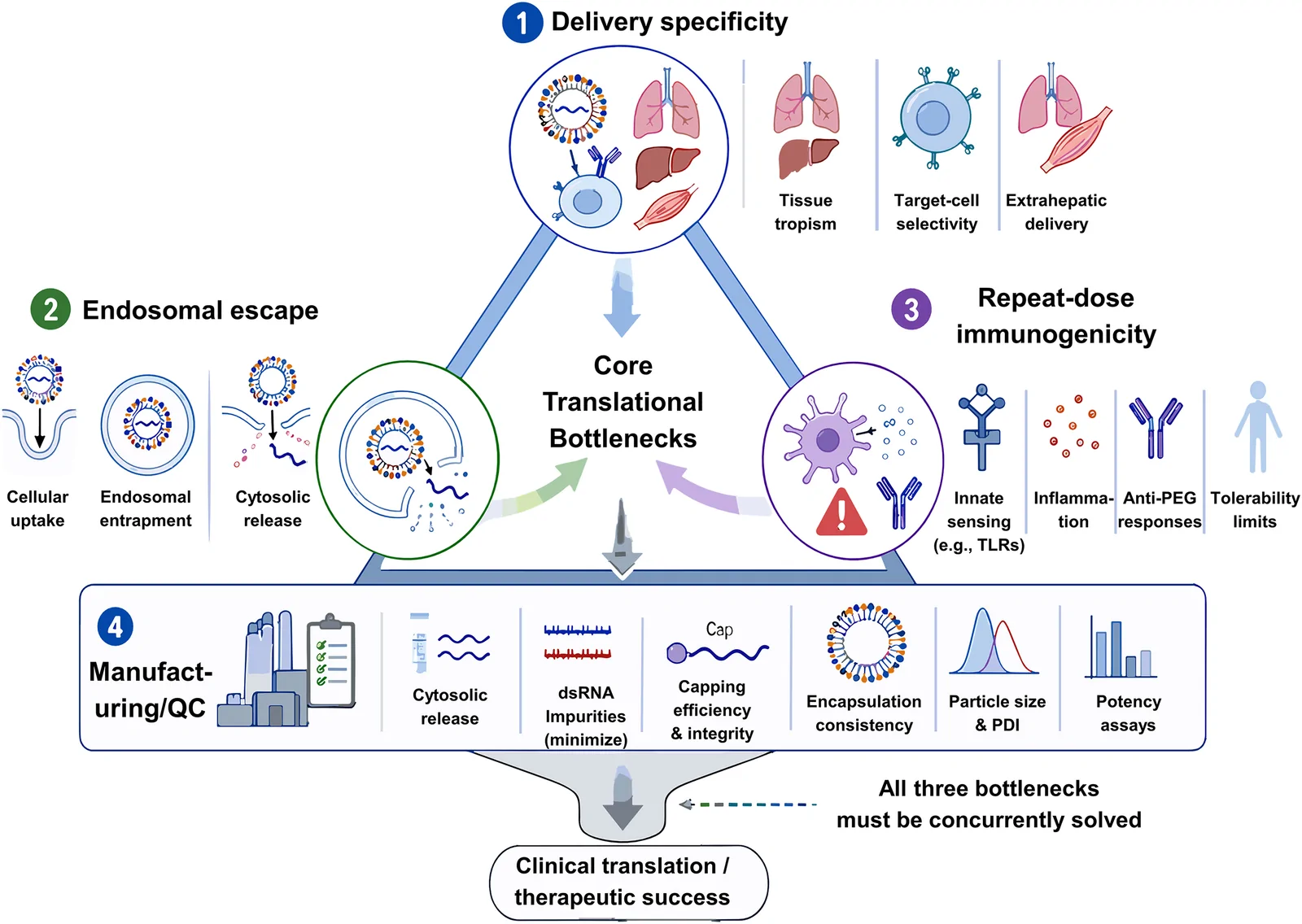
Translational bottleneck triangle in mRNA medicine The schematic highlights the interconnected barriers of delivery specificity, endosomal escape, repeat-dose immunogenicity, and manufacturing quality control that collectively determine clinical translation. This schematic figure was prepared based on author-provided conceptual sketches and scientific layout, using FigureLab for scientific illustration and manual author editing.

### Targeted delivery and biodistribution control

The central translational challenge in mRNA medicine is increasingly the ability to achieve functional expression in disease-relevant cells while limiting expression in off-target tissues. Primary *in vivo* studies demonstrate that nanoparticle composition can substantially alter the cellular and organ distribution of systemically administered mRNA. High-throughput functional screening identified LNPs capable of delivering mRNA and genome-editing components to splenic endothelial cells rather than predominantly to hepatocytes.^55^ SORT nanoparticles subsequently demonstrated that modification of LNP composition can redirect functional mRNA delivery and CRISPR-Cas activity among the liver, lung, and spleen,^56^ while mechanistic studies linked this organ tropism to changes in nanoparticle biodistribution, apparent pKa, and serum-protein interactions.^57^ Other primary studies have demonstrated lung-selective mRNA delivery after systemic administration^60^ and receptor-directed mRNA delivery to hematopoietic stem cells *in vivo*.^64^ Collectively, these findings indicate that extrahepatic delivery is not a single formulation problem but depends on matching nanoparticle composition, targeting strategy, administration route, and target cell biology. Accordingly, a more informative translational benchmark is not simply whether a formulation accumulates in an extrahepatic organ but whether it reproducibly produces functional expression in the disease-relevant cell population at clinically feasible doses while limiting off-target expression.

### Endosomal escape as the central intracellular bottleneck

Cellular uptake alone is not sufficient for productive mRNA delivery. Direct analysis of multiple LNP-mRNA formulations showed that total cellular uptake did not predict functional delivery; mRNA could accumulate in endosomal compartments that were poorly productive for release, whereas escape events were preferentially associated with early and recycling endosomal compartments.^127^ Structure-activity studies of ionizable lipids further linked improved mRNA pharmacology in rodents and non-human primates to enhanced endosomal escape.^29^ More recent live-cell and super-resolution imaging of mRNA- and small interfering RNA (siRNA)-loaded LNPs identified multiple post-uptake barriers to cytosolic delivery, including incomplete RNA release and segregation of ionizable lipid and RNA cargo during endosomal sorting.^128^ Together, these primary studies support endosomal escape as a major determinant of productive intracellular RNA delivery rather than cellular uptake alone. Importantly, maximizing membrane disruption is not necessarily optimal. Endosomal membrane damage caused by LNPs can recruit galectins and promote inflammatory signaling, whereas ionizable lipids that generate smaller, endosomal sorting complexes required for transport (ESCRT)-repairable membrane lesions can maintain high mRNA expression with reduced inflammation.^129^ Thus, the relevant design objective is not simply to maximize endosomal escape but to increase productive cytosolic release while limiting damaging membrane perturbation.

### Innate immune activation and repeat-dose feasibility

Innate immune activation remains a double-edged property of mRNA systems. In vaccination, innate sensing can contribute to adaptive immune priming,^32^ whereas repeated therapeutic dosing introduces additional formulation- and carrier-dependent constraints. Primary studies show that PEG-lipid structure and abundance can affect dose-responsive mRNA expression across repeated administrations^130^ and that anti-PEG antibodies can activate innate immune cells and mediate hypersensitivity responses to PEGylated nanomedicines.^131^ Several complementary strategies are being investigated to reduce the dependence of repeat-dose mRNA formulations on conventional PEG-lipids.^132^^,^^133^^,^^134^^,^^135^^,^^136^ One approach retains PEG during nanoparticle formulation but limits its persistence after administration: carboxylesterase-cleavable PEG-lipids have been used to promote PEG detachment *in vivo* and reduce carrier-directed immune memory during repeated mRNA vaccination in mice.^136^ More direct replacement strategies have incorporated polysarcosine lipids, which maintained or increased mRNA delivery efficiency in ALC-0315- and SM-102-based LNPs while showing comparable *in vivo* safety profiles to PEG-containing formulations.^134^ Poly(2-ethyl-2-oxazoline) lipids have likewise supported mRNA delivery to hepatic and extrahepatic cell populations while reducing bloodstream clearance and anti-stealth-lipid IgM responses relative to PEG-lipids in mice.^135^ Zwitterionic polymer-lipid conjugates have been used to replace PEG-lipids completely in nebulizable mRNA-LNPs, improving formulation stability during aerosolization and enabling repeated pulmonary administration without detectable lung inflammation in mouse models.^132^ Carbohydrate-derived glycolipids provide another PEG-free strategy; maltoheptaose-based glycolipid LNPs maintained mRNA delivery efficiency over repeated administrations while reducing anti-PEG antibody production and increasing splenic accumulation.^133^ Nevertheless, replacement of PEG is not a single-variable solution because the identity, structure, and abundance of the surface lipid influence repeat-dose performance, whereas de-PEGylation kinetics can alter blood clearance, organ deposition, and cell-type-specific delivery.^130^^,^^137^ These alternatives therefore remain preclinical platform-specific solutions rather than universally validated replacements, and their development must balance reduced carrier immunogenicity against particle stability, biodistribution, cellular uptake, and productive intracellular delivery.^132^^,^^133^^,^^134^^,^^135^ Accordingly, repeat-dose compatibility should no longer be treated as a secondary formulation detail; it is a central determinant of which diseases are realistically addressable by mRNA therapeutics.^130^^,^^131^

### Manufacturing, purity, and analytical quality control

The manufacturing challenge is to achieve reproducible functional quality at clinical and commercial scale. Experimental studies have shown that capping chemistry, RNA purification, and the manufacturing process can materially affect translation and innate immune activation.^10^^,^^11^^,^^37^ Critical quality attributes therefore span transcript identity and integrity, capping and poly(A) characteristics, process-related impurities, formulation attributes, and biological potency. Recent analytical advances such as long-read sequencing-based vaccine analysis and engineered T7 RNA polymerases that reduce immunostimulatory byproducts illustrate how quality control is becoming a major scientific field in its own right rather than a downstream manufacturing afterthought.^138^^,^^139^

### Disease selection and clinical positioning

A mature mRNA field must also be selective. The clinical examples reviewed above indicate that mRNA is not automatically the best modality for every disease or every protein target. Its strongest use cases are settings in which transient expression is sufficient or advantageous or in which short-lived RNA exposure can generate a longer-lived protein product or durable downstream effect, as illustrated by mRNA-1944, mRNA-3927, and NTLA-2001.^13^^,^^14^^,^^15^ The liver-directed protein-replacement therapy mRNA-3927, which encodes the PCCA and PCCB subunits of propionyl-CoA carboxylase for propionic acidemia, is compelling because periodic restoration of enzyme activity may be sufficient to modify metabolic disease biology without genomic integration.^15^ The mRNA-encoded antibody therapy mRNA-1944, which transiently produces the anti-chikungunya monoclonal antibody CHKV-24 *in vivo*, illustrates a different advantage by shifting biologic production from *ex vivo* manufacturing to the treated individual.^13^ Nexiguran ziclumeran (NTLA-2001), which delivers Cas9 mRNA and a TTR-targeting guide RNA for TTR amyloidosis, represents another distinct use case in which transient nuclease expression is coupled to a durable genomic edit.^14^^,^^96^^,^^97^ The future of the field will therefore depend not only on better chemistry but also on choosing diseases whose therapeutic logic genuinely matches what mRNA does best.^13^^,^^14^^,^^15^

### AI-enabled sequence engineering and precision nanodelivery

AI and machine learning should be viewed as enabling tools rather than as replacements for biological experimentation. In mRNA design, deep learning frameworks are already being used to optimize coding sequences and UTRs for improved translation.^140^ In parallel, machine-learning-guided chemistry is accelerating the discovery of ionizable lipids and helping prioritize candidates for *in vivo* testing more efficiently than purely empirical screening workflows.^141^ The most realistic near-term role of AI is therefore iterative acceleration: narrowing sequence space, improving formulation screening, predicting structure-function relationships, and guiding higher-value experiments in both transcript design and nanoparticle engineering.^140^^,^^141^ It is best framed as an emerging layer of optimization for precision nanodelivery and expression control, not as an autonomous solution to the core biological barriers of mRNA therapeutics.^140^^,^^141^

## Conclusion

Taken together, the primary clinical evidence reviewed here supports understanding mRNA as a therapeutic protein-expression platform rather than as a vaccine-only technology.^13^^,^^14^^,^^15^ Primary clinical studies now demonstrate therapeutic use beyond vaccination in protein replacement, inhaled gene complementation, regenerative signaling, antibody expression, local tumor immunomodulation, genome editing, and transient cell engineering.^13^^,^^14^^,^^15^^,^^66^^,^^67^^,^^92^^,^^104^ The next phase of progress will depend less on whether an mRNA can express a protein and more on whether expression can be controlled in the relevant cells and tissues, at an appropriate magnitude and duration, and with acceptable repeat-dose performance. In that sense, future progress will depend not only on sequence design but also on precise delivery, productive cytosolic release, reproducible manufacturing, and selection of diseases in which transient or programmable protein expression offers a genuine therapeutic advantage.

More broadly, the clinical and preclinical evidence reviewed across these applications indicates that therapeutic performance cannot be predicted from the encoded sequence alone. Different applications impose distinct requirements for delivery efficiency, tissue accessibility, expression kinetics, repeat-dose feasibility, and safety, as illustrated by clinical studies of antibody expression, protein replacement, and genome editing.^13^^,^^14^^,^^15^ Platform matching therefore requires that RNA architecture, formulation strategy, administration route, target tissue, and expression profile be aligned with the biological requirements of the intended application. The resulting framework favors application-specific integration of molecular design, delivery engineering, manufacturing control, and disease selection rather than a universal mRNA solution. This platform-matching approach constitutes the organizing framework of the present review.

## Acknowledgments

This study was supported by research fund from 10.13039/501100002457Chosun University, 2026 (K1000702004). Ethics approval was not applicable to this review article.

## Author contributions

K.-S.L. and J.-Y.P. contributed to literature review, conceptualization, and manuscript drafting. C.-U.K. supervised the work, contributed to conceptual development, and revised the manuscript. All authors reviewed and approved the final manuscript.

## Declaration of interests

All authors attest they meet the ICMJE criteria for authorship.

## Declaration of generative AI and AI-assisted technologies in the manuscript preparation process

During the preparation of this work, the authors used ChatGPT (OpenAI) to assist with language editing, text refinement, and improving manuscript readability and FigureLab as a dedicated scientific illustration tool to convert author-provided conceptual sketches and scientific layouts into polished schematic artwork. The authors reviewed, edited, and revised all AI-assisted content as needed and take full responsibility for the content of the published article. No AI tool was used to generate, alter, or interpret original experimental data or primary research images, and no general-purpose generative AI image tool was used to create the graphical abstract *de novo*.
